# The utility of copy number variation (CNV) in studies of hypertension-related left ventricular hypertrophy (LVH): rationale, potential and challenges

**DOI:** 10.1186/1755-8166-6-8

**Published:** 2013-03-01

**Authors:** Hoh BoonPeng, Khalid Yusoff

**Affiliations:** 1 Institute of Medical Molecular Biotechnology, Faculty of Medicine, Universiti Teknologi MARA, Sungai Buloh Campus, Jalan Hospital, Sungai, Buloh, 47000, Malaysia; 2 Faculty of Medicine, Universiti Teknologi MARA, Selayang Campus, Jalan Prima 7, 68100 Batu Caves, Selangor, Malaysia

**Keywords:** Copy number variation, Genetic susceptibility, Hypertension, Left ventricular hypertrophy

## Abstract

The ultimate goal of human genetics is to understand the role of genome variation in elucidating human traits and diseases. Besides single nucleotide polymorphism (SNP), copy number variation (CNV), defined as gains or losses of a DNA segment larger than 1 kb, has recently emerged as an important tool in understanding heritable source of human genomic differences. It has been shown to contribute to genetic susceptibility of various common and complex diseases. Despite a handful of publications, its role in cardiovascular diseases remains largely unknown. Here, we deliberate on the currently available technologies for CNV detection. The possible utility and the potential roles of CNV in exploring the mechanisms of cardiac remodeling in hypertension will also be addressed. Finally, we discuss the challenges for investigations of CNV in cardiovascular diseases and its possible implications in diagnosis of hypertension-related left ventricular hypertrophy (LVH).

## Introduction

Genetic variation in human genome exists in different forms, ranging from large, cytogenetically visible chromosomal alterations to submicroscopic variations including structural changes involving insertion/deletion of large DNA segment, segmental duplication, copy number changes and inversion, to the molecular level of presence/absence of transposable elements, variable number of tandem repeat (VNTR), and changes of a single base pair known as single nucleotide polymorphism (SNP).

SNP was thought to be the predominant form of variation in human genome and accounted for the majority of phenotypic variability [1]. A number of genome-wide association studies (GWAS) had reported the relation of these common variants in human disease susceptibility. For instance, the Wellcome Trust Case Control Consortium (WTCCC) involving 17,000 samples, studied seven common diseases namely, hypertension, Crohn’s disease, type I diabetes, type II diabetes, coronary heart disease, rheumatoid arthritis and bipolar disorder [2]. Many other GWASes have identified genetic associations of a wide range of common and complex diseases [eg. [3-7]. To date, the number of reported GWAS is still growing exponentially (http://www.genome.gov/gwastudies/).

Recently, an alternative form of genetic variation has gained much interest namely, Copy Number Variation (CNV). Since the first report [8,9], it has emerged as an important genetic marker in addition to SNP. It is conceivable that CNV will be taking its place alongside SNP in various genetic studies in the near future. Hence it is timely to review the potential role of CNV in understanding complex polygenic conditions in particular cardiovascular diseases, including hypertension and its complications. This review discusses the possible utility of CNV in delineating the mechanisms of cardiac remodeling in hypertension and its challenges in translation to clinical practice.

### Copy Number Variation (CNV)

**Figure 1:**
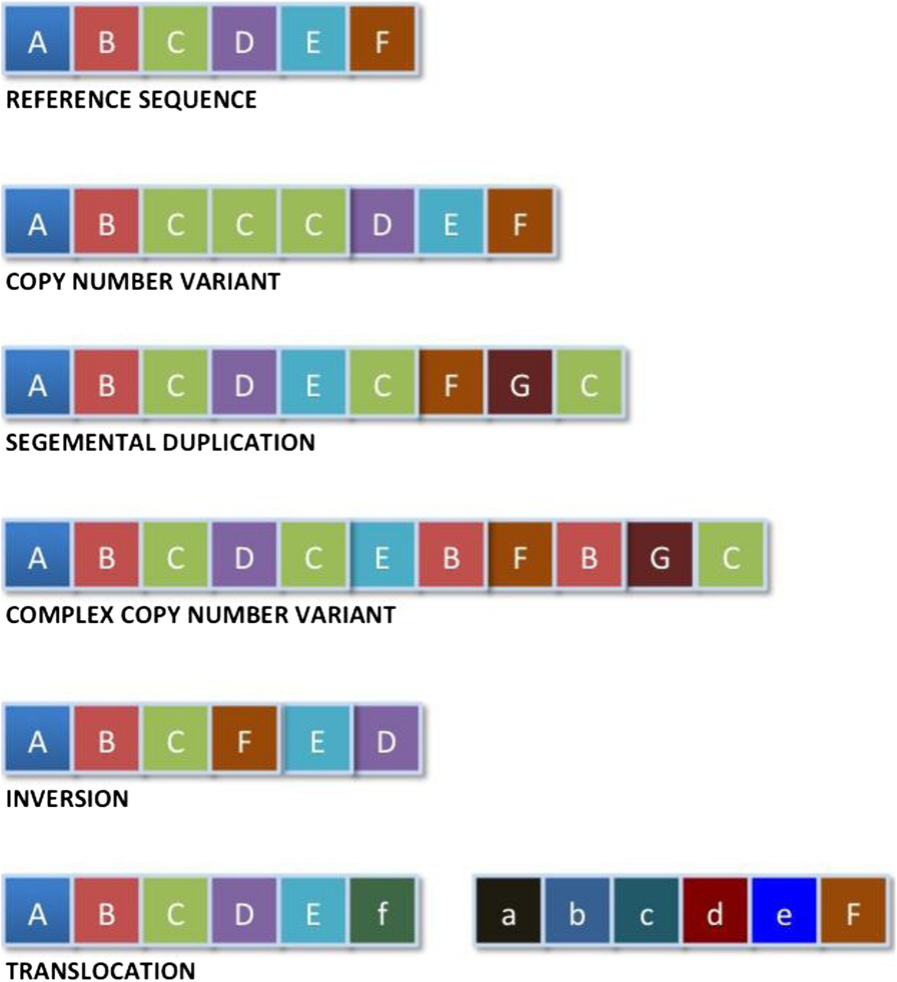
Genomic alterations involving large segments of DNA resulting in formation of structural variation and copy number variation.

CNV is recognized as a form of structural variation, involving changes of copy number of a large segment of DNA (>1 Kb) composed of duplications, deletions, and complex multi-site variants introduced by non-allelic homologous recombination [10,11]. It is by and large the most prevalent type of structural variation identified to date [12]. Figure 1 illustrates the types of genomic alterations resulting in the formation of structural variation and copy number variations in the genome.

Despite technological limitations in CNV discovery, this genetic marker is estimated to cover ~12 – 15% of the human genome [13,14]. Interestingly, there is a significant relationship between the CNV regions and gene content - a substantial proportion of CNVs are found at the gene-enriched regions in particular those “environmental sensitive” genes, which influence the responses to environmental stimuli [15,16]. Indeed a significant association either with gene expression variability [17], or the important regulatory sequences that are situated far apart from the actual target gene [12].

Initially, CNV was thought to be pathogenic to rare genomic disorders [12,18,19]. However, this variability appears virtually in all “phenotypically normal” individuals reported to date [8,9,20-22]. It has been attributed to a number of susceptibility of common and complex diseases [23-28], neuropsychiatric diseases [29-33], and notably, cardiovascular diseases [34,35].

### Technology for CNV detection and analysis

CNV can be detected and analyzed by different technologies, both at the genome-wide scale and the locus specific level. Details of these technologies have been well described [36,37].

At the genome-wide level, the following methods are predominantly in use currently:

i) array-Comparative Genomic Hybridization (aCGH). This assay uses an array of probes where differentially labeled test and reference DNA are jointly hybridized to the array [36,37]. Detection of CNV is based on the intensity ratio of the labeled probes. Two major technologies are available for aCGH. Agilent Technologies commercializes high-density arrays, up to 1 million probes; while NimbleGen Technology produces arrays with 2.1 million probes.

ii) SNP array. Although this assay was originally developed for investigations of single nucleotide variation, it is increasingly being used to mine CNVs, based on the probe intensity information obtained. In SNP array, no reference DNA is being labeled. Instead, CNV is identified via comparing the probe intensities of the test DNA with different individuals. Two major platforms are available in the industry. Affymetrix produces high-density “hybrid” arrays, accommodating 1.8 million of SNP and CNV probes. Whilst the Illumina produces up to 5 million SNP probes in a single array, the highest density in the microarray technology thus far. Whilst SNP array provides both information of SNP and CNV with a higher density, aCGH has a better signal accuracy. Another advantage of SNP array/“hybrid” array over aCGH is its ability to identify the runs of homozygosity (ROH), providing a more informative analysis. ROH is defined as a large DNA segment (typically > 1 Mb) with the presence of uninterrupted homozygosity resulting from mutational events such as uniparental disomy (UPD), hemizygous deletion and loss of heterozygosity (LOH) [38]. Often it is challenging to distinguish between the ROH with a copy number deletion and with the presence of both homozygous alleles. SNP array has the ability to differentiate the two types of ROH via inspection of the genotype calls and probe signal intensity ratio simultaneously. Several studies on ROH and complex diseases have been reported recently [39,40].

iii) *Fluorescent* in situ *Hybridization* (FISH). This approach was initially used to detect various syndromic diseases involving larger scale chromosomal alternations. It hybridizes fluorescently labeled probes to the chromosomal regions showing high sequence complementary, subsequently visualized under fluorescent microscopy. However, one major limitation of FISH in CNV analysis is its low resolution, making it incapable of accurately detecting any CNV smaller than 5 Mb [36], thus rendering it inappropriate for study of common and complex disease like LVH.

At the locus specific level, technologies being utilized in CNV quantification of a particular candidate gene including, but not restricted to, Multiplex Ligation-dependent Probe Amplification (MLPA), quantitative PCR (qPCR), and Paralogue Ratio Test (PRT). MLPA utilizes the principle of multiplex PCR and ligation, simultaneously detecting CNV in multiple regions in a single reaction, based on its PCR ligated products. The copy number of the sample is the measurement of the intensity of the product relative to control DNA [41]. qPCR is the commonest and simplest assay for CNV analysis. It measures the fluorescence signals of the targeted sequence relative to a reference gene (e.g. FOXP2 and TERT) [41]. PRT uses a single primer pair to amplify precisely two products, one from the targeted CNV region and the other from a single copy reference locus [42]. Our experience suggests that PRT was more accurate in determining copy number calls compared to other PCR based assays.

The introduction of high throughput next generation sequencing has significantly increased the resolution and sensitivity of CNV detection. However, the cost of experiment is considerably higher and may not be affordable by most laboratories.

### Left Ventricular Hypertrophy (LVH)

LVH is an independent risk factor for the development of clinical events such as heart failure, cardiac arrhythmias, stroke and cardiovascular mortality [43,44]. A recent report suggested the prevalence of LVH at 36% globally, based on echocardiographic studies [45]. In Malaysia, the prevalence of LVH was reported to be 24% based on echocardiography [46]. Initially it is a physiological response to hemodynamic and/or biomechanical stress, as in hypertension [47,48]. Later the hypertrophy is mal-adaptive and counterproductive, and impacts adversely on the prognosis. LVH remains a highly frequent indicator for cardiac damage among hypertensive patients [45]. The development of LVH in a patient with hypertension worsens the prognosis of the patient. This condition has a complex multifactorial and polygenic basis for its pathogenesis.

Control of hypertension and reversal of hypertension-related LVH is possible, resulting in improved prognosis. Identification of patients at risk for developing hypertension-related LVH may lead to early interventions to prevent its development, hence avoiding its sequelae and complications. This may further improve the prognosis and help increase adherence to treatment among these patients. Thus early diagnosis and improved understanding on the pathogenesis of LVH may lead to more effective therapeutic strategies [49].

### Genetics of LVH

**Figure 2:**
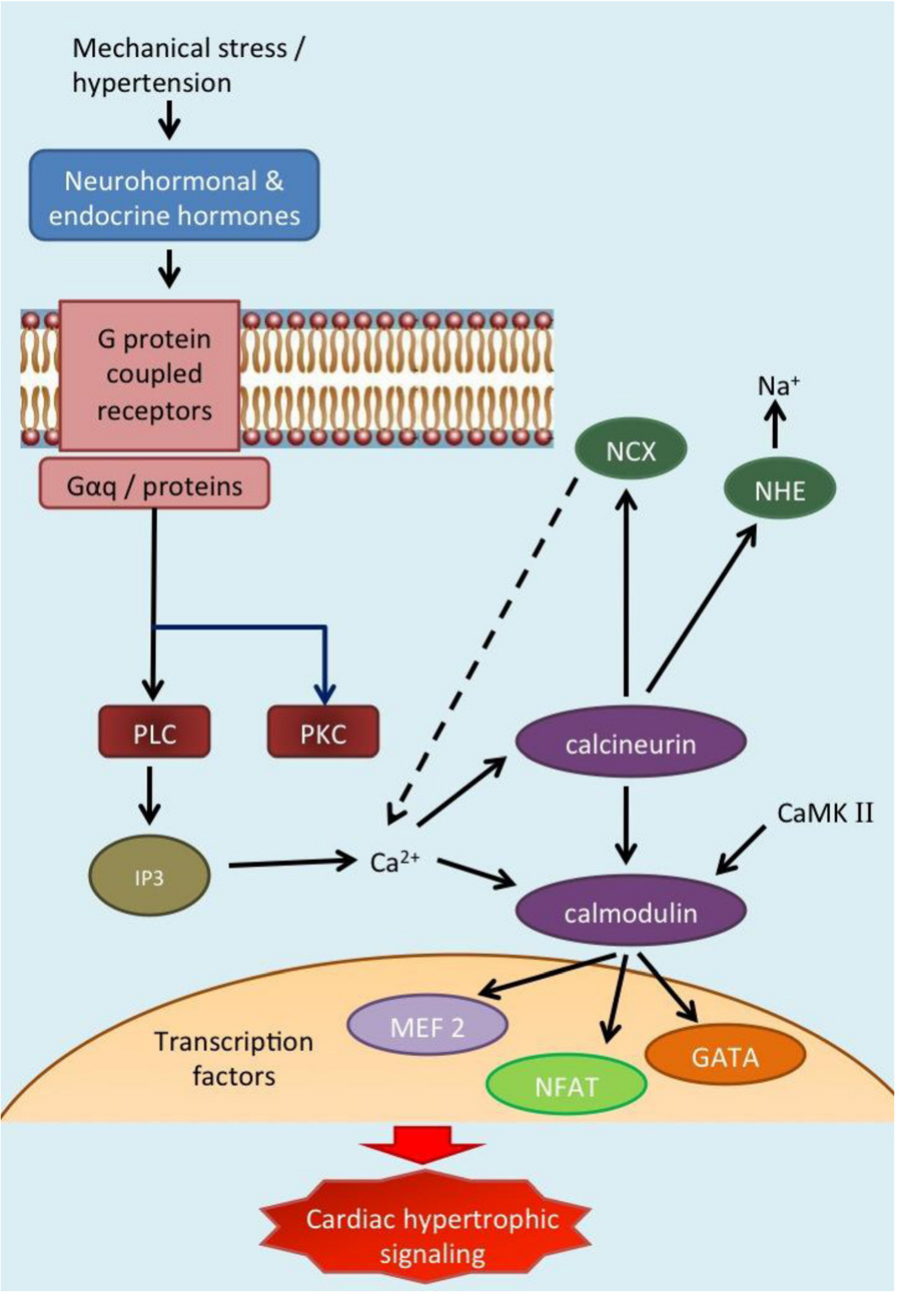
Molecular pathways of left ventricular (LVH) pathogenesis.

The molecular and biochemical pathways of LVH pathogenesis have been well described [43,44,47,50-52]. Briefly, mechanical input on the myocytes is transduced into biochemical event by initiation of signaling at the cell membrane. Neurohormonal and endocrine hormones (e.gAng II, EDN I, IGF I) bind to Angiotensin II type 1 and type 2, β-adrenegic and endothelin receptors which coupled to heteromeric Gq-proteins, induce phospholipase C (PLC) and protein kinase C (PKC) activation and subsequently production of inositol 1,4,5-triphosphate [43,44,50] (Figure 2). Downstream, the release of Ca^2+^ triggers the calcineurin-calmodulin pathways, which activates various transcription factors including nucleus factor activated T cell (NFAT) and myocyte enhancer factor 2 (MEF2), JNKs, and GATAs [44,47,50,52]. The calcineurin pathway is also closely connected to several ion channel pathways, including Na^+^/Ca^2+^-exchanger (NCX), and Na/H–exchanger (NHE) pathways. Activation of NHE pathway results in increased Na^+^, thus increasing the Ca^2+^ influx via NCX pathway, and initiating cardiac hypertrophy signaling. At the same time, NFAT transcription factor, which was triggered by calcineurin, interacts with the ANP/BNP pathways [43]. Other suggested pathways include, but not limited to, the PPAR, metalloproteinase (MMP) and mitogen-activated protein kinase (MAPK). The combination of these mechanisms conspires to initiate cardiac hypertrophy. These complex networks interacting with each other, together with pleiotropic and/or epistasis effects of the molecular pathways provide a substantial challenge to the efforts of mapping the causative genes.

LVH can be reversed by specific anti-hypertensive agents. Losartan, an angiotensin receptor blocker, has shown clinical benefit of this reversal beyond controlling the blood pressure [53]. Although a number of candidate gene association studies suggested the involvement of Renal Angiotensin System (RAS) in LVH pathogenesis, this association remains inconclusive, as results were not consistently replicated [54,55]. Notably, a genome-wide association study (GWAS) from the HyperGEN Study showed no significant association of RAS genes with LVH, instead calcineurin related genes seemed responsible for the development of LVH [55,56]. However when the same study cohort (HyperGEN) was tested on candidate genes ACE and AGT variants, significant association with LV mass was detected [57]. On top of that, a meta-GWAS published recently found no significant association signals of any of the RAS related gene with LV mass and function [58]; whilst Arnett et al. [59] reported that the NCAM1 was associated with LV wall thickness. Further, Gallego-Delgado et al. [60] showed that RAS mediated genes were not up- nor down-regulated in a transcriptomic study. These findings further strengthen the evidence that the influence of RAS to LVH is most likely indirect. The mechanism of action of Losartan could possibly be explained thus: it selectively blocks Angiotensin II receptor I (AGTRI) but allowing continued signaling via Angiotensin II Receptor II (AGTRII) which reduces cellular proliferation, fibrosis and Matrix Metalloproteinase (MMP) activities by normalizing the calcineurin activity and the DNA binding of the NFAT signaling, yet increases apoptosis in cardiac cells [61,62]. Collectively, whether a strategy specific to reduce LVH provides additional clinical advantage or simply to reverse the complication by controlling blood pressure is debatable.

Several observations can be made based on the genetic studies of LVH:

i) Selection of candidate genes solely based on an understanding of the biochemical roles of encoded proteins is not comprehensive thus may lead to inappropriate selection of candidate genes or SNPs. Therefore, an unbiased approach (often with multiple hypotheses) such as genome-wide screening [63] is always recommended to study complex traits such as hypertension-related LVH. Further, interactions between genes and environment as well as haplotype analyses have been insufficient thus potential biological influence of a particular candidate gene may have been masked [64].

ii) Precise phenotyping. The first generation of GWAS completed by the Wellcome Trust Case–control Consortium (WTCCC) failed to achieve a genome-wide significant level in hypertension [2] and up to 25% of the population based “controls” were misclassified [65]. This diluted the statistical effects thus biased towards null [65,66]. Therefore, obtaining a “clean” and standardized case and control group to one particular trait (example in this case, LV wall thickness, or LV mass) from a particular homogenous cohort is essential. In order to increase the power of detection, one should consider deriving samples from an extreme phenotype distribution [65], for instance the 10^th^ percentile versus the 90^th^ percentile of the LV wall thickness measurement [67,68].

iii) Common traits (eg. blood pressure and LV wall or LV mass) may be controlled by many genes or loci (in a single or several pathophysiological pathways), each accounts a small portion of additive genetic effects on the phenotype. These common variants do not contribute to immediate effect to the deleterious phenotypes, rather to late onset common and complex diseases [69].

iv) “Missing heritability”. The fact that the “unexplained reason(s)” of the relatively small fraction of individual disease risk (modest odd ratio of ~1.1-1.5) suggest that there are missing puzzles when explaining the trait heritability [70,71]. Instead, the less common variants (0.5-5%) or rare variants (<0.5%) with stronger effect may play an equal role as of common variants, if not greater. Nonetheless, these variants are often too rare to be detected by common molecular tools to prove statistical evidence of association [69].

v) The influence of other forms of genome variations such as CNV. The potential of this variability is yet to be fully explored, therefore its impact on common diseases such as hypertension-related LVH is largely unknown. There has been arguments though, that common CNV is typable by tagSNP, therefore they contribute limited risk factor to disease susceptibility [12,72]. However, the complex, segmental duplicated and the rare *de novo* CNVs are not counted - at least not with the current genotyping platform (except for next generation sequencing) because they normally do not follow the Mendelian inheritance. In addition, CNV allelic size is often not “taggable” by these SNPs [12]. Typical examples are shown in a number of diseases [23,24,27,30-32,73].

### CNV and the susceptibility of hypertension-related LVH

Association between CNV and cardiovascular diseases has not been widely investigated hence less understood, except that those reviewed by Pollex and Hegele [74], which mainly described the monogenic disorders of cardiovascular diseases, and recently on familial dilated cardiomyopathy [34]. While studies on gene copy number of LPA gene in familial hypercholestrolemia (FH), atherosclerosis and coronary artery disease [19] were performed, the influence of CNV in non-familial cardiovascular diseases in particular hypertension-related LVH has not been widely reported. It is possible that CNV may be involved in pathogenesis of LVH via its potential biological effects on the candidate genes (ie regulating the gene dosage) in the respective pathways. Essentially, complex diseases like hypertension and other cardiovascular diseases, together with additive environmental factors, might be more prone to a “softer” form of variation such as CNVs, which alters the gene dosage (or regulations) without disrupting the functions. We have listed here some of the selected candidate genes and their molecular/biochemical pathways (mainly RAS, calcineurin-calmodulin, MAPK and other above mentioned related signaling pathways) which are believed to be involved in the pathogenesis of LVH. They often overlap with known or common CNV regions. The search was done through the Database of Genomic Variants (DGV; http://projects.tcag.ca/variation/) (date: 17^th^ May 2012) (Table 1).

**Table 1:** Candidate genes and their functions in hypertensive LHV pathogenesis

**Gene**	**Gene map locus**	**Gene function**
**RENAL-ANGIOTENSIN SYSTEM/G-COUPLED PROTEIN RECEPTORS:**		
ATP6AP2	Xp11.4	Muscular smooth muscle contraction. Renin and prorenin cellular receptor. May mediate renin-dependent cellular responses by activating ERK1 and ERK2. By increasing the catalytic efficiency of renin in AGT/angiotensinogen conversion to angiotensin I, it may also play a role in the renin-angiotensin system (RAS).
**Estrogen receptor signaling:**		
EGFR	7p11.2	Receptor binding to epidermal growth factor. Required by AngII to mediate ERK activation thus plays a critical role in the LVH induced by Ang II.
EGF	4q25	Shedding of heparin-binding EGF by ADAM12, induce hypertrophic signaling via EGFR activation downstream through small G proteins and the MAPK pathway.
ADAM12	10q26.2	Involved in skeletal muscle regeneration, specifically at the onset of cell fusion and macrophage-derived giant cells (MGC) and osteoclast formation from mononuclear precursors. Plays a centre role in cardiac hypertrophy by interacting with HB-EGF.
ERBB2	17q21.1	A member of the epidermal growth factor (EGF) receptor family of receptor tyrosine kinases. Essential in cardiac development. Involved in the EGFR signaling that drives many cellular responses, including changes in gene expression, cytoskeletal rearrangement, anti-apoptosis and increased cell proliferation.
ITGB2 (Beta-integrin)	21q22.3	Involved in cell adhesion as well as cell-surface mediated signalling. Links the extracellular matrix to the intracelluar cytoskeleton. Stretch sensor. Deletion of this gene/protein leads to cardiac pathology. Gene expression profile is upregulated in ERbeta knockout mice with cardiac hypertrophy.
ITGB1BP2 (Melusin)	Xq13.1	Integrin interacting protein. Sensor of mechanical stress in cardiac myocytes. An essential component for the phosphorylation (inactivation) of GSK3-beta. Deletion of this gene leads to cardiac pathology.
**CALCINEURIN-CALMODULIN Ca**^**2+ **^**Signaling:**		
PPP3CA (calcineurin A-alpha)	4q24	Calcium dependent, calmodulin stimulated protein phosphatase.Ca2+/calmodulin binding.
CALM1 (calmodulin 1)	14q32.11	Mediates the control of large number of enzymes and other proteins by Ca^2+^. Involved in a genetic pathway that regulates the centrosome cycle and progression via cytokinesis.
CALM3 (calmodulin 3)	19q13.2	Together with CALM1 and CALM2, these calmodulin genes give rise to five transcripts that are present in most tissues. This gene may be specifically and differently regulated during cardiac cell proliferation and/or hypertrophy.
CAMK2B (calmodulin kinase)	7p14.3	Calmodulin/Ca^2+^ signaling. At basal Ca^2+^ levels, CaMKs are maintained in a dormant state through autoinhibition. Increase in Ca^2+^ levels allows calmodulin to relieve this autoinhibition and activate the kinase activity.
**Ion channel pathway:**		Sodium-Calcium exchange pathway
SLC8A1 (NCX1)	2p22.1	
SLC9A2 (NHE)	2q12.1	Sodium-Hydrogen exchange pathway
KCNB2	8q13.3	Regulate the smooth muscle contraction by controlling the influx of Ca^2+^ through voltage-gated Ca^2+^ channels
**NFAT SIGNALING & OTHERS TRANSCRIPTION FACTORS:**		
NFATC3	16q22.1	Required in muscle cell, and heart development, as well as smooth muscle differentiation. Rapid nuclear exit of NFATC is thought to be onemechanism by which cells distinguish between sustained and transient calcium signals.
ILF3	19p13.2	Involved in the NFAT transcription signaling. A subunit of the nuclear factor of activated T-cells (NFAT).
ITPR3@ IPR3	6p21.31	Inositol 1,4,5-triphosphate receptor type 3. Involved in mediating the release of intracellular calcium.
GATA4	8p23.1	Transcription factors that regulate genes critical for myocardial differentiation and function, regulates hypertrophic gene expression.
MCIP-1 (RCAN1)	21q22.12	Modulatory calcineurin interacting protein. Serves as calcineurin–regulatory protein that inhibit calcineurin when over expressed.
MEF2A	15q26.3	Transcription factor of cardiac hypertrophy cascade. Involved in the activation of numerous growth factor- and stress-induced genes. Mediates cellular functions in skeletal and cardiac muscle development.
MAPK3	16p11.2	Act as a signaling cascade that regulates various cellular processes including proliferation, differentiation and cell cycle progression in response to a variety of extracellular signals.
JAK2	9p24.1	Involved in a specific subset of cytokine receptor signaling pathways. Uponreceptor activation JAKs phosphorylate the transcription factors known as STATs and initiate the JAK-STATsignaling pathway.
CTNNB1 (Beta-catenin)	3p22.1	Cadherin-associated protein. Part of a complex of proteins that constitute adherens junctions. Involved in the regulation of cell adhesion. Predominantly localized to the cell membrane, and is part of E-cadherin/catenin adhesion complexes which are believed to couple cadherins to the actin cytoskeleton. Structural changes of the extracellular matrix in LVH are significantly modulated by B-catenin associated signaling pathways.

Analysis of the CNV with hypertensive LVH though, should not limit to only those particular “gene-enriched” CNVs. Studies have shown that CNVs may have impact on disease susceptibility through their effects on non-transcribed domains that regulate gene expression at a distance [10,16]. On the other hand, the possible influence of rare or *de novo* CNV should not be ignored in particular some of the extreme cases. Typical successful example is shown in thoracic aortic aneurysms and dissections [34].

### Implications of CNV in clinical practice of LVH

CNV has opened up potentials for both clinical cases and laboratory medicine. Its path may lead to the advancement for laboratory tests, which are used to be time consuming, labor intensive, expensive and at times difficult to interpret the result. Together with echocardiography and ECG examinations [75,76], CNV and/or SNP based genotyping could enrich the information for diagnosis, providing a more accurate and efficient detection tool, of which early intervention may be beneficial to hypertensive patients at risk of developing LVH. A successful example is the study reported by Wang et al. [77]. Coupled with the CNV of LPA gene and the SNP identified, this study managed to increase the detection rate of Familial Hypercholestrolemia (FH) up to 86.2%. To the best of our knowledge, this study has yet to be replicated; but if successful, this result could be useful to design for FH diagnosis, and consequently be developed as a biomarker if functionally validated. The genetic profiles obtained may be useful to estimate disease risk, eg LVH, for the individual hypertensive patient.

In essence, large segment CNV alternations often involve numerous neighboring genes, thus shedding the light on the understanding of syndromology ie wide spectrum of variations and inconsistencies of phenotypic features [12]. Hypertension-related LVH, expressing a wide range of phenotypes, could be explained via gene mapping with CNV. Though, the applications of CNV in medical decision-making should be considered with caution at this present stage, due to the fact that frequencies of occurrence may vary in a particular population from another.

Despite hunting for causative variants, the primary value of genome-wide studies – either SNPs or CNVs mapping – is to provide an etiological connection between biochemical pathways and disease, thus providing novel insights into the disease mechanisms. Consequently, knowledge of the disease pathways can be translated into strategies for prevention, diagnosis and therapy including drug development, new treatment and diagnostic approaches. Essentially candidate CNVs identified in both genome-wide scale, and locus specific assays, should be functionally validated and replicated in an independent cohort. Eventually specific tests of the particular CNV(s) could be developed in clinical practice.

### Major challenges of CNV in cardiovascular diseases

Investigations of CNV in complex diseases, while tempting, are still in its developing stage. Several challenges are noted currently and expected to resolve in the near future, especially with the availability of the massively parallel high throughput sequencing:

i) Lack of replication in CNV callings and reference samples for comparison of results. Multiple commercial arrays available for CNV detection, each with different resolutions and analytic algorithms, bias towards the advantage of its own technology. This leads to a poor replication when reporting CNV, and lack of a standardized reference sample set further complicates the analysis efforts [78].

ii) Limitation of the current array based technology (copy number calls). Due to limitation of the chemistry, the array-based platforms only allow CNV detection of gain or loss of two copies (CN = 0 – 4). Thus, analysis of multiple or complex CNV has always been a challenge.

In view of the above mentioned difficulties, CNV is usually called by three algorithms, and the consensus of at least two is defined as “stringent” CNV call [36]. In addition, validation approaches with other techniques are always recommended to confirm the CNV detected, for instance, qPCR, MLPA, paralogue ratio test (PRT), CGH and FISH (for large CNVs) [13,79,80].

iii) Identification of precise breakpoint is a common issue of CNV study. Often, the non-identical, but overlapping copy number alleles is being assigned into one category, or being misinterpreted as the same allele. This will definitely affect tests of association between variation and disease, as different breakpoints may result in different biological consequences. DNA sequencing of the region of interest may resolve this problem. However, it is laborious in Sanger sequencing for such purpose [81]; the cost for next-generation sequencing is expected to be lower in the future though.

iv) Careful interpretation of the complex CNV data in both research and medical decision-making is essential especially when dealing with complex diseases. How CNV results are applied to research or medical decision-making needs to be considered according to the circumstances where it is observed especially with the awareness of interactions between single nucleotide alterations and CNVs [12]. Unexplained familial disorders should be revised with the consideration of the presence of above mentioned potential technical limitations. Potential challenge between genomic observation and clinical implication could be raised when complex conditions like cardiovascular diseases require a shift of mindset to approaches like rare CNVs.

v) The need for an established database, both for “healthy” populations (such as Database for Genomic Variants, DGV), or disease cohort (such as DECIPHER) will be essential, cataloging the information of genic content of the variant segment [12,82]. This would help to narrow down the disease diagnosis by excluding those non-pathogenic, and focus on the disease-based CNVs.

vi) Cost consideration versus selection of technology. Effective running cost and ease of assay typing have always been the major hindrance for clinicians in CNV analysis. Although the cost for an array-based global CNV screening has dropped, it is unaffordable by most patients especially those from the lower income community. Although locus specific CNV genotyping assays are more cost effective, lower resolution and limited information obtained (as discussed in the earlier section) from such assays is a drawback. Therefore, comprehensive CNV mapping and identification of pathogenic CNV in LVH is needed to ensure that the findings be translated into clinical practice in the most cost effective manner.

## Conclusion

Molecular cardiological research with CNV provides ample possibilities for the development of more cardiac-specific pharmaceutical interventions, which could be tailor made to the pathology and genetic make-up of the individual patient. The application of CNV in complex diseases will continue to grow while array based technology will remain as the mainstay in the next future. Ongoing development of technological and sophisticated statistical tools in CNV analysis should warrant the extensive investigation of the genetics of LVH. However, the growth of this research area is still in its infancy stage and far from translating into medical implication, thus efficacy of such an approach should be carefully interpreted.

## Competing interests

The authors declare that they have no competing interests.

## Authors’ contributions

HBP and KY wrote and edited the paper. All authors read and approved the final manuscript.
